# Implant displacement to the maxillary sinus– a retrospective multicenter cohort study and a management protocol

**DOI:** 10.1186/s40729-025-00629-3

**Published:** 2025-06-06

**Authors:** Daniel Muchnik, Gavriel Chaushu, Eli Rosenfeld, Shaked Adut, Aiman Elmograbi, Meir Debecco, Amir Laviv, Daya Masri

**Affiliations:** 1 Department of Oral and Maxillofacial Surgery, Rabin Medical Center— Beilinson Hospital, Petach Tikva, 49414 Israel; 2 Department of Oral and Maxillofacial Surgery, The Maurice and Gabriela Goldschleger, School of Dental Medicine, Tel Aviv University, Tel Aviv, 69978 Israel; 3 Department of Otolaryngology, Head and Neck Surgery, Rabin Medical Center—Beilinson Hospital, Petach Tikva, 49414 Israel; 4 Department of Oral and Maxillofacial Surgery, Samson Assuta Medical Center, Ashdod, 7747629 Israel

**Keywords:** Dental implants, Maxillary sinus, Sinusitis, Oroantral fistula

## Abstract

**Purpose:**

This study aims to investigate the potential complication of implant displacement into the maxillary sinus, exploring its etiology and various management strategies, while proposing a systematic approach for clinicians to effectively address this evolving complication.

**Materials and methods:**

This retrospective multi-center cohort study evaluated patients with dental implant migration into the maxillary sinus treated between 2010 and 2023 at two Israeli medical centers. Data included demographics, symptoms, clinical findings, and treatment modalities.

**Results:**

32 patients (mean age: 62.3 years) were analyzed, with a notable incidence of sinusitis and oroantral fistulas. 91% required surgical intervention, predominantly Functional Endoscopic Sinus Surgery (63%). The majority of displacements occurred during the implantation process, often correlating with clinical symptoms.

**Conclusion:**

As shown in our study, displacement of implants into maxillary sinus, often leads to sinusitis and oroantral fistula. A proposed treatment algorithm emphasizes surgical intervention, particularly Functional Endoscopic Sinus Surgery, based on symptoms and clinical signs.

## Background

Dental implants have become a common solution for restoring upper posterior teeth. However, several factors must be addressed to achieve successful long-term rehabilitation, including poor bone quality of the maxilla, pneumatization of the maxillary sinus, and alveolar bone resorption in edentulous areas. A critical factor influencing the success of the implantation procedure is primary stability, which prevent micromovements, facilitate clot adherence and the beginning of bony healing process around implant. Late migration of the implant into the sinus can occur due to loss of osseointegration, which may happen during the exposure procedure, after loading due to peri-implantitis or unfavourable distribution of occlusal forces. Other risk factors for displacement of dental implants into the maxillary sinus have been suggested to include inadequate drilling protocol, and insufficient surgical experience [1, 2]. 

This potential complication in oral implantology must be considered by clinicians [3, 4]. Research indicates that dental implants are frequently encountered foreign bodies within the maxillary sinus [5, 6]. Such complications can result in serious sequelae, including the development of oroantral fistula (OAF), maxillary sinusitis, and, in rare instances, orbital cellulitis or intracranial infections [7–9]. 

Various therapeutic approaches have been proposed, ranging from observation without removal to several surgical techniques [10, 11]. These methods have evolved considerably over the years, with the “Caldwell-Luc” and lateral sinus approaches being the most documented in the literature [12–14]. In the early 2000s, functional endoscopic sinus surgery (FESS) emerged as a significant technique in this area [14, 15]. 

Most existing literature has primarily reported individual cases or small series, making it challenging to draw comprehensive conclusions. This study aims to investigate the potential complication of implant displacement into the maxillary sinus, exploring its etiology and various management strategies, while proposing a systematic approach for clinicians to effectively address this evolving complication.

## Materials and methods

### Aim

Examine implant displacement into the maxillary sinus, its causes, management strategies, and propose a systematic approach for clinicians to handle this complication.

### Study design

This study was conducted as a retrospective multi-center cohort study.

### Study group

The study involved patients diagnosed with migration of dental implants into the maxillary sinus who sought treatment at the Department of Oral and Maxillofacial Surgery from 2010 to 2023 at two medical centers in Israel: Rabin Medical Center, Beilinson Campus, Petah Tikva, and Assuta Medical Center, Ashdod.

### Inclusion criteria

Participants included those referred to the maxillofacial surgery departments of the aforementioned medical centers with a confirmed diagnosis of dental implant migration to the maxillary sinus.

### Exclusion criteria

Patients were excluded if they did not have a “floating” implant in the maxillary sinus, lacked panoramic or CBCT radiographs, if demographic records were inadequate. Patients with a zygomatic implant which was present on the side where the displacement occurred, were also excluded.

### Data collection

Data were gathered from patient records using a structured form, compiled from the hospitals’ medical records, and organized in Microsoft Excel.

Demographic information recorded included age, gender, ASA classification, and smoking history. The reason for hospital referral was also documented, detailing whether it was due to dentist referral, any symptoms (rhinologic or dental), incidental findings (without symptoms), or pre-prosthetic needs. Rhinologic symptoms encompassed headache, smell disturbances, and nasal discharge or congestion, while dental symptoms included oral pus discharge, pain around the implant site, intra/extraoral swelling, or fluid passage between the oral and nasal cavity.

Sinusitis was diagnosed according to the international multidisciplinary consensus statement on the diagnosis of odontogenic sinusitis (ODS) [16]. This consensus highlighted the need for both otolaryngologists and dental specialists to recognize sinusitis based on specific clinical features, ensuring that patients are appropriately referred for diagnosis confirmation.

The features that raise suspicion of sinusitis include:

1. Unilateral sinus symptoms, such as foul odor, loss of smell, posterior nasal drainage, anterior nasal drainage, nasal obstruction, and facial pressure.
2. Unilateral maxillary sinus opacification seen on CT scans.

To confirm sinusitis, an otolaryngologist should perform nasal endoscopy, which may reveal purulence, edema, or polyps.

Data on the timing of implant displacement to the sinus were categorized as either immediate (during insertion or removal of the implant) or late (occurring during the osseointegration period or subsequent exposure procedure). If information regarding the timing was unavailable, it was classified as NA (Not Available). The status of whether the implant was placed immediately after extraction was also noted, with missing information marked as NA. In addition, whether or not sinus elevation was performed prior to implant placement, was recorded.

All patient symptoms and clinical signs were documented, including pain, pus discharge (oral or nasal), the presence of OAF and diagnosis of sinusitis. Patients referred to ENT when exhibited rhinological signs which is suspicious for sinusitis (facial pain/pressure, foul smell, any nasal discharge, congestion or blockage or symptoms and/or obstruction of the osteo-meatal complex (OMC). No nasal signs or symptoms were reported by the patients before the implantation procedure.

Treatment modalities were categorized as surgical or non-surgical. Surgical retrieval methods included techniques such as FESS, Caldwell-Luc approach, lateral sinus approach, and transcrestal approach. Non-surgical treatments encompassed no treatment, symptomatic relief, or antibiotics. Any complications during treatment or in the postoperative period were also recorded. If OAF was present, the closure technique was investigated. Timing for removal was categorized based on whether it occurred within one year of implant displacement or after one year.

Radiographic data were derived from panoramic, Cone Beam Computed Tomography (CBCT) and Multidetector Computed Tomography (MDCT) images. The choice of which CT to perform was primarily determined by clinical signs and symptoms. Patients without rhinologic complaints were referred for CBCT, while those with rhinologic issues and/or OMC obstruction on CBCT were referred for MDCT. Documented data included whether the OMC was obstructed, the thickness of the sinus membrane (considered pathological > 2 mm), sinus floor thickness in the area of implant displacement, which maxillary sinus the implant migrated into, and whether other paranasal sinuses were affected. Additionally, if available, the length of the displaced implant was measured on CBCT in both coronal and sagittal views, utilizing an in-program ruler.

The final proposed treatment algorithm was developed based on the data collected and the analysis of clinical sequelae and radiographic findings.

### Statistical analyses

Statistical analyses included descriptive statistics arranged in Microsoft Excel’s tables. All relevant study data (number of cases for each category, average, and standard deviation -SD, were calculated using Excel’s in-program functions).

## Results

### Demographic data

**Fig. 1:**
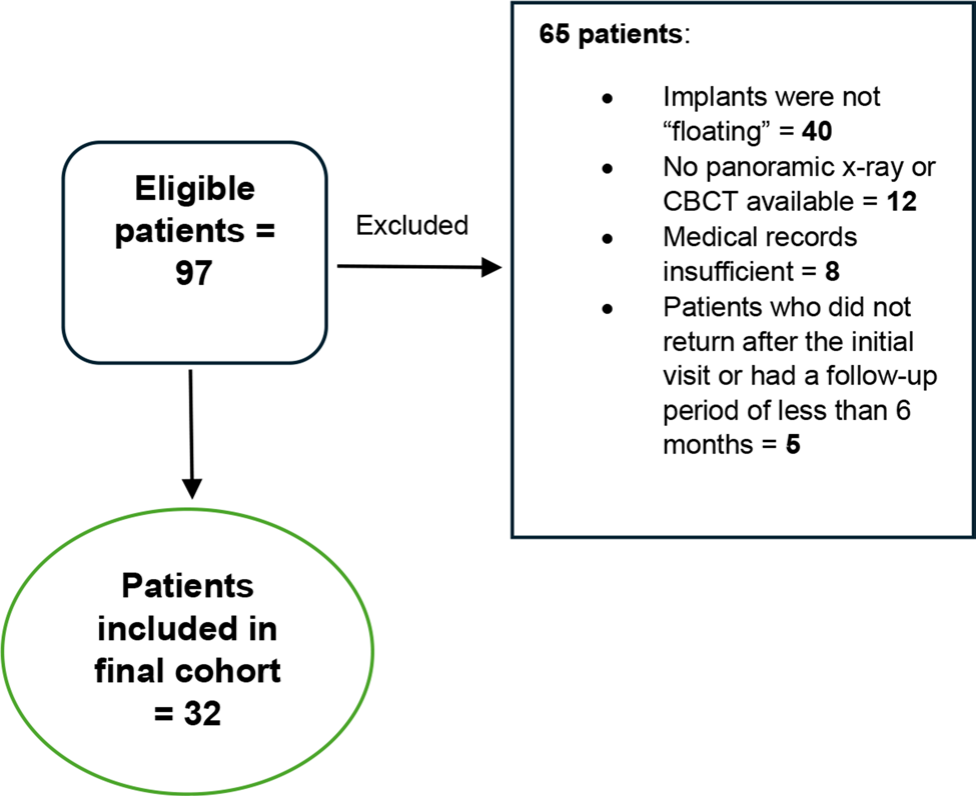
Patient inclusion & exclusion process

This study included a total of 32 patients who were diagnosed with displaced implants into the maxillary sinus (Table 1). Data regarding patients who were excluded summarized in Fig. 1.

The mean age was 62.3 years (ranging from 19 to 86 years, SD = 14.7). The patient demographic consisted of 18 males and 14 females, and the American Society of Anaesthesiologists (ASA) classification showed that no patients exceeded ASA 3, indicating a relatively low surgical risk within the cohort. Among the participants, 10 patients (31%) had a history of smoking.

### Background for implant displacement

Patients were referred for various reasons. The predominant admission reasons included dental and rhinologic symptoms (12 patients), pre-prosthetic evaluations (9 patients), dentist referral after displacement (8 patients) and incidental findings (3 patients). Notably, 36% (12 patients) reported symptoms related to the migrated implant, including 8 patients with rhinologic symptoms and 4 with dental symptoms. Pus and pain were the most frequently observed clinical signs, present in 16 patients (50%) and 10 patients (31%), respectively.

### Timing of implant displacement

In 8 out of the 15 patients (53%) for whom data was available, implant migration occurred during the implantation process, while in another 4 patients (27%), it happened during the exposure phase. Rest of the implants migrated during osseointegration period (between implant placement & exposure procedure).

### Sinusitis and OMC obstruction

Sinusitis was observed in 16 patients (50%). Radiological signs of thickened membrane were seen in this subgroup (100%) and ostium obstruction presented in CT in 13 patients (81%).

### Implant length and sinus membrane thickness

The mean implant length was 10.8 mm (out of the available information), and the average sinus floor thickness measured 2.2 mm, while the maximal thickness reached 3.6 mm. Only 9 patients (28%) had prior history of sinus floor elevation.

### Surgical intervention

In terms of surgical interventions for managing complications (Table 2), FESS was performed in 20 patients (63%), while 4 patients underwent Caldwell-Luc procedures, 4 (9%) had lateral approaches and 2 underwent trans-crestal approach (6%); In 2 patients no surgical treatment needed, while in 1 of them (3%) the implant was discharged from nasal complex during sneezing. Twelve patients exhibited OAF, with specific surgical methods utilized for their closure, including buccal advancement flaps, buccal fat pad and palatal rotation flap.

### Timing of implant removal

Among the patients received surgical treatment (30 patients), implant removal was conducted with a mean time to removal documented at less than one year in 17 patients (57%) and greater than one year in 13 patients (43%).

## Discussion

Dental implants have become a prevalent solution for patients with missing teeth. This procedure has become a widespread procedure and is performed by many dentists and oral surgeons. As the frequency of implant placement, especially in the posterior maxilla, is rapidly increasing, the number of complications such as the displacement of implants to the maxillary sinus will probably also rise.

### Signs and symptoms of implant migration to the maxillary sinus

In the present study, 12 out of 32 patients (37.5%) referred with clinical symptoms, while 8 of them (67%) suffered from rhinologic symptoms and 4 (33%) suffered from dental symptoms. Another 12 (37.5%) patients were admitted with migrated dental implant to the maxillary sinus as coincidental finding or for prosthetic reason, while 5 of them (42%) had clinical and/or radiological signs of sinusitis. Other 8 patients out of the total cohort (25%) were referred by the dentist after displacement in different time-frames which will be discussed later. Only 3 of them (37.5%) diagnosed with sinusitis, 3 had pain and OAF (37.5%). This data indicates that a significant number of patients may remain asymptomatic and seek treatment either as a random discovery or for future prosthetic needs.

Migration of implants into the maxillary sinus may irritate the sinus lining or block the OMC, which could lead to sinusitis. While the development of sinusitis may be postponed for several months or even years, prior studies have indicated that not all patients with displaced implants show clinical symptoms or significant radiographic pathological changes. In another study detailing 9 cases, 3 patients reported sinus infections, whereas the remaining 6 did not show any symptoms [17]. Chiapasco et al. noted in his study that 13 out of 21 patients were asymptomatic and only 8 patients sought for medical care due to sinusitis symptoms or OAF [11]. Petruson et al. also stated that the existence of dental implants in the maxillary sinus does not automatically lead to sinus-related issues [18]. Possible explanation he mentioned is that production of nitric oxide inside the sinus may prevent infections around implants [19]. 

### Etiology and timing of implant migration

Regarding the potential causes and risk factors for implant migration, we examined sinus floor thickness, implant length, and prior sinus floor elevation. According to dental history and CBCT, only 9 patients (28%) had a previous history of sinus floor elevation. All patients had less than 4 mm of residual bone height (averaging 2 mm) around the area of implant displacement, while the average implant length was 10.7 mm. In a study by Sgaramella et al. only 6 out of 21 patients (29%) had undergone a sinus lift procedure before implant placement, and in almost all cases, no traces of bone graft material were found at the time of examination [4]. The same conclusions were drawn in another study by Manor et al. which showed implant migration occurred mainly in cases of bone deficiency and lack of sufficient residual bone for osseointegration [20]. This data suggests that inadequate treatment planning or poorly executed bone augmentation significantly contribute to the risk of implant displacement into the sinus. It underscores the importance of achieving primary stability, which is crucial for a successful surgical outcome. 

In the present study in 8 out of 15 patients (53%) the implant migration occurred during implant placement procedure. As highlighted in earlier studies, obtaining adequate primary implant stability during simultaneous sinus elevation can be challenging, with a minimum residual bone height of 4 to 5 mm recommended [21, 22]. If the implant is unstable during placement, it may be necessary to remove it and carry out the grafting and implant placement in two separate stages. When the bone height is less than 5 mm, primary stability is at risk, leading to the traditional recommendation of performing a sinus lift and bone augmentation, with implant placement occurring 4–6 months later as a second stage. However, it is now possible to perform a sinus lift and bone augmentation with implant placement as a single stage even when the subantral bone is below 5 mm, provided certain conditions are met and there is significant surgical expertise. Additionally, bone quality should be considered, as it may necessitate underdrilling and the use of a wider implant. However, a key requirement for a one-stage approach is achieving initial implant stability [22, 23]. In 4 out of 15 patients (27%) the migration occurred during exposure procedure, which may be explained by failure of implant osteointegration and/or application of excessive forces during the procedure or in attempt to remove the non-integrated implant. Three implants migrated during osteointegration period (20%) and in other 17 patients data about the timing of implant displacement was not available, but we hypothesize that at least some of them were displaced during late period after exposure, which may also occur due to excessive loading forces, or progressive bone loss and peri-implantitis [24]. In this study, no information was available regarding the clinical experience of the clinician who performed the implant surgery, or treatment plan and surgical protocol that was executed before admission to the hospital. In summary, the primary factors related to the etiology of implant displacement identified in our study are linked to inadequate bone support and the subsequent challenges in achieving primary stability. This is evidenced by the insufficient bone height observed in our patient cohort, the inadequate or absence of prior bone augmentation procedures, and the fact that displacement occurred at the time of implantation in the majority of cases.

### Clinical and radiological signs

Membrane thickening was observed in 88% of cases (28 out of 32 patients), and ostium obstruction was seen in 16 cases (50%), of which 13 patients were diagnosed with sinusitis. OAF were present in 12 patients (37.5%), with 50% (6 patients) experiencing obstruction of the OMC. The presence of a thickened membrane (over 2 mm) may be attributed to a foreign body reaction, local inflammation, or even infection resulting from the displaced implant and/or augmented sinus floor. When a migrated implant disrupts the anatomy of the Schneiderian membrane, it can impair mucociliary function, hindering ventilation and drainage, and allowing bacteria and viruses to access the OMC. The OMC may become obstructed for various anatomical and functional reasons, contributing to the development of sinusitis [25]. All these factors may subsequently lead to the formation of OAF, as indicated in previous studies [11, 26]. Chiapasco et al. reported that 7 out of 13 patients with sinusitis had ostium obstruction (54%), a lower prevalence than noted in our study (13 out of 16 patients, 81%). They also found that 19 out of 27 patients diagnosed with oroantral communication (70%), a figure which is higher comparable to our study (12 patients, 37.5%). Manor et al. Also reports higher prevalence of OAF (83%) [20]. This could be attributed to the timing of definitive treatment, which may be longer in other studies. In the present study, all OAFs that developed in patients with sinusitis were associated with obstructed OMC (5 cases), leading to impaired drainage. Conversely, the OAFs that developed in patients without sinusitis may have arisen due to the position of the displaced implant along with the presence of dense inflammatory tissue that facilitated OAF formation.

### Treatment modalities

A total of 29 patients were treated surgically (91%), with FESS being employed in most cases (69%), followed by Caldwell-Luc procedure (14%), lateral approach (10%), and trans-crestal approach (7%). In one patient, the implant dislodged spontaneously through the nose during sneezing, despite her experiencing rhinologic symptoms and being scheduled for surgical intervention. In two patients (6%), no treatment was necessary as they exhibited no clinical symptoms or signs, and there were no intended prosthetic treatment plans. These patients were informed about the importance of strict follow-up. Few studies suggested that displaced dental implants in the maxillary sinus can lead to chronic maxillary sinusitis, necessitating surgical intervention even in asymptomatic patients [27, 28]. This perspective is also supported by the recent meta-analysis, which found that only 3.8% of cases were simply observed [2]. Observation may be considered in the absence of clinical symptoms or upon the patient’s request. It may also be indicated when the surgical removal of the intra-sinus implant poses risks due to its proximity to the orbital floor. In our study, 29 out of 32 patients (91%) underwent surgical intervention for implant removal, consistent with findings from other reports. Although some studies have indicated long-term follow-ups (greater than 12 months) without irritation of the sinus mucosa, such cases are rare, and patients must be adequately informed about the long-term risks of infection [1, 29–31]. 

**Fig. 2:**
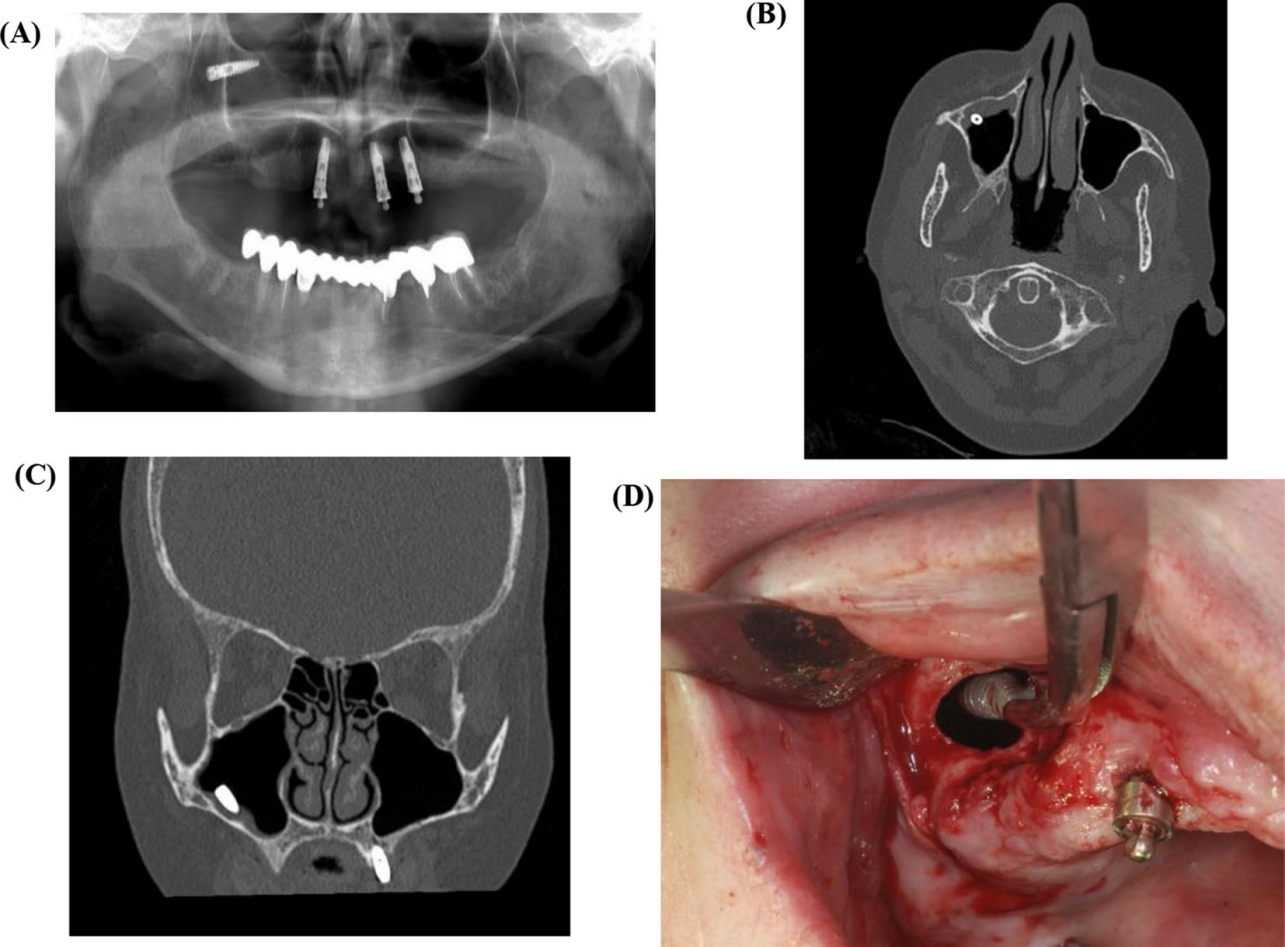
Displaced implant removal using lateral sinus approach. (**A**) Pre-operative panoramic x-ray, (**B**). Pre-operative CT in axial and (**C**) coronal view’s demonstrating the implant inside the right maxillary sinus with membrane thickening, (**D**) Removal of the implant through the “lateral approach”

**Fig. 3:**
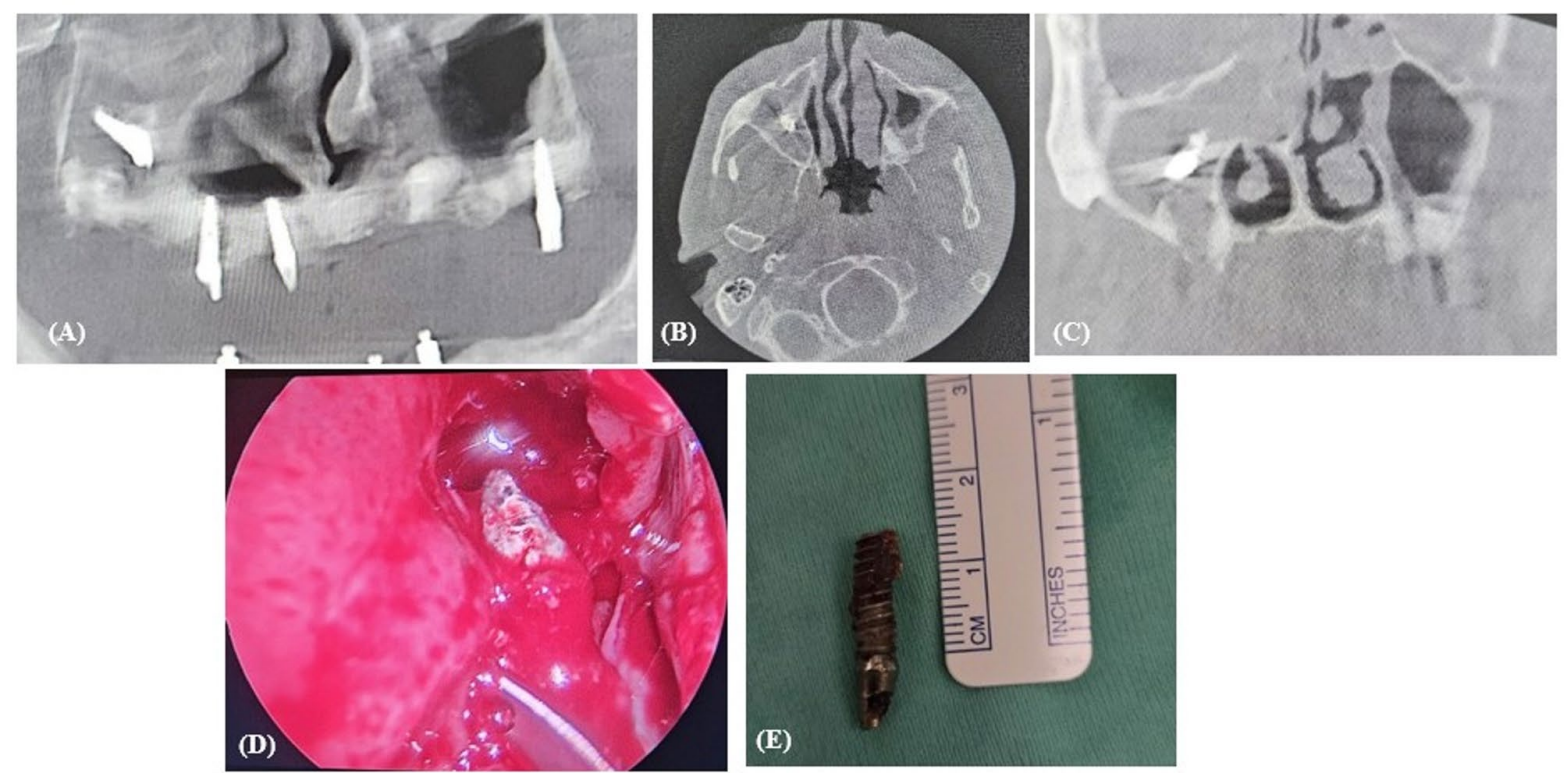
Displaced implant removal using FESS. (**A**) Pre-operative panoramic x-ray; (**B**) Pre-operative CT in axial view demonstrating the implant inside the right maxillary sinus with sinus opacification; (**C**) Pre-operative CT in coronal view demonstrating the implant inside the right maxillary sinus with OMC obstruction; (**D**)Removal of the implant through FESS; (**E**) Implant after removal

The traditional method for treating sinus pathologies and removing foreign bodies has been the Caldwell-Luc surgery. This procedure involves creating a bony window near the canine fossa to access the maxillary sinus via the anterior wall, including performing an inferior meatal antrostomy. While this technique was commonly used in the early 20th century, it has become less frequently indicated in light of developments such as the lateral approach (Fig. 2) and FESS (Fig. 3), which are less invasive. Common complications associated with Caldwell-Luc surgery included recurrent nasal obstruction, facial asymmetry, facial numbness, OAF, wound dehiscence, dacryocystitis, devitalized teeth, recurrent sinusitis, and recurrent polyposis [13, 30].

Tatum introduced less invasive intra-oral lateral sinus and trans-crestal approaches, which eliminate the need for additional inferior antrostomy and are now favored for sinus lifts, augmentations, and the removal of foreign bodies, usually leading to fewer complications [32]. However, they do not address cases with OMC obstruction.

FESS has become increasingly popular as a treatment for sinus pathologies and for retrieving foreign bodies. This approach facilitates the removal of displaced implants, addresses paranasal sinusitis, and restores proper patency of the natural maxillary ostium through a minimally invasive technique [33, 34]. One of the primary side effects reported from FESS is the formation of synechiae due to scar formation between the inferior turbinate and nasal septum, which appear to have minimal clinical implications [33]. This evolution in surgical techniques is reflected in our study, where FESS (69%) was the most common approach, particularly in recent years (2019 and later), whereas the Caldwell-Luc procedure was mainly employed earlier. Nevertheless, FESS cannot address OAFs, which require additional intra-oral surgical approaches. Small OACs (less than 5 mm) can typically close on their own after the formation of a blood clot, while larger defects or those present for more than three weeks often require surgical intervention [35]. For small OACs that occur immediately after implant retrieval, applying a collagen plug and suturing the mucosa may be adequate. In contrast, larger defects or chronic communications that have epithelialized (OAFs) will likely necessitate local flaps, such as buccal advancement flaps, buccal fat pad flaps, palatal flaps, and possibly bone grafts and membranes, depending on the size and location of the OAF [36]. A multidisciplinary approach involving ENT and maxillofacial teams is essential in these cases. If an OAF is present and FESS is indicated due to clinical or radiological signs of sinusitis, it is advisable to address both conditions in the same surgical procedure. If an OAF exists without the requirement for sinus drainage, it can be treated during a transoral implant retrieval, or if removal is done via FESS, it is preferable to do it simultaneously for the patient’s convenience.

### Treatment algorithm

**Fig. 4:**
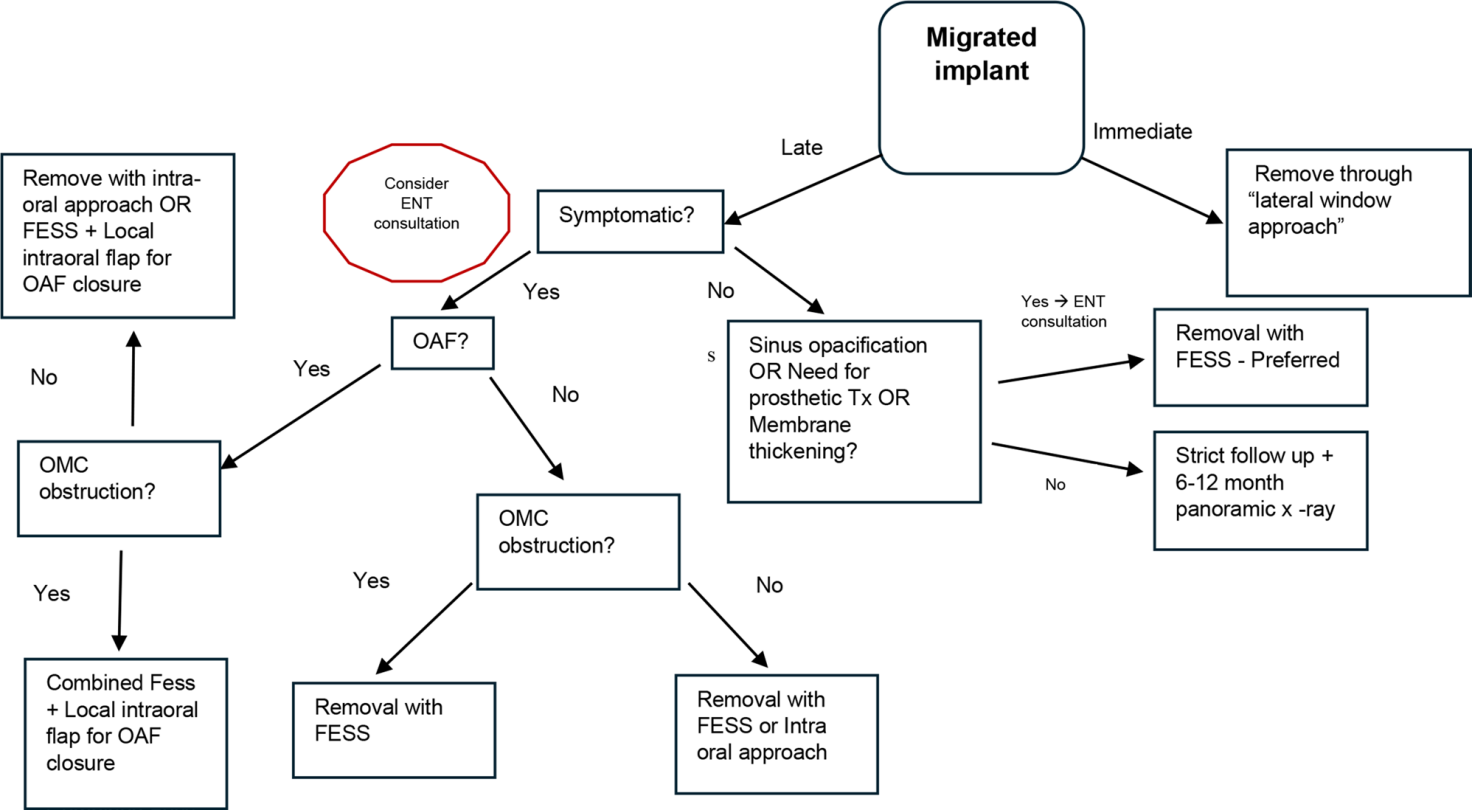
Proposed treatment protocol for patient diagnosed with migrated implant to the maxillary sinus (**OMC**– Osteo-meatal complex, **OAF–** Oroantral fistula)

Our proposed treatment algorithm, based on the data presented in our study, depicted in Fig. 4.

A comprehensive initial assessment is crucial, encompassing a detailed medical history and clinical examination to identify dental or nasal signs/symptoms (pain, pus, nasal obstruction, swelling, headache, and OAF presence). 3D Imaging, is then necessary to visualize implant location, membrane thickness, sinus opacification, and OMC obstruction. Treatment decisions are guided by clinical presentation, while immediate implant removal is recommended, if possible.

Asymptomatic patients and no OAF, undergo risk stratification based on patient preference, prosthetic needs, membrane thickness, and sinus opacification which warrants surgical removal - FESS preferred. High-risk patients (those requiring pre-prosthetic intervention, significant membrane thickening, and/or sinus opacification) should undergo surgical removal to mitigate long-term risks. Low-risk patients warrant close monitoring with regular imaging (6–12 months) and strict clinical follow up. If any clinical signs or symptoms arise, or if there are any changes observed in radiological imaging, an intervention is warranted.

Symptomatic patients require surgical intervention: FESS is the primary approach for sinusitis (with or without OAF), potentially combined with intraoral flaps for OAF closure. In the absence of sinusitis, but with OAF, a transoral approach or FESS (depending on implant location) is used for removal of implant, followed by fistula repair. OMC obstruction necessitates surgical intervention to restore drainage– through FESS.

Whenever a suspicion for sinusitis rise according clinical or radiological data, a referral to otolaryngologist is mandatory. In other cases, if there is no OAF, the implant may also be removed by FESS and a consultation with otolaryngologist is recommended.

We suggest that any transoral procedure be conducted by a maxillofacial surgeon or a highly skilled dental surgeon who are experienced with these types of procedures. Additionally, FESS should be carried out by an otolaryngologist.

Postoperative management involves close monitoring for complications, antibiotic prophylaxis where appropriate, and patient education.

### Strengths and limitations

A significant strength of this study is its multicenter approach, which allows for a more comprehensive analysis of 32 cases over a 13-year period, providing valuable insights into the etiology and management of implant displacement into the maxillary sinus. The proposed treatment protocol, informed by a thorough examination of diverse clinical scenarios, offers a systematic approach that can enhance the standard of care for practitioners addressing this complication.

Despite its contribution to understanding dental implant displacement, this study has some limitations. The retrospective design may introduce selection bias, as it relies on available medical records, potentially omitting critical data from patients who did not seek treatment.

## Conclusion

The study highlights the increasing prevalence of implant displacement to maxillary sinus, emphasizing the need for improved preventative strategies and evidence-based treatment protocols. Analysis of 32 cases revealed that displacement can occur during implant placement, exposure, or osseointegration, often leading to sinusitis and OAF.

A proposed treatment algorithm, guided by the presence or absence of symptoms and sinusitis, advocates for surgical intervention (FESS being the preferred method) in most cases, while emphasizing the importance of thorough initial assessment, including advanced imaging, and careful consideration of individual patient factors.

Although this retrospective study cannot establish definitive causality, the results underscore the need for improved surgical techniques and meticulous pre-operative planning to reduce the incidence of this complication and to guide clinicians towards optimal management strategies. Further prospective studies are warranted to validate the proposed algorithm and assess the long-term outcomes of different treatment modalities.

**Table 1:** Study cohort data and characteristics

*N*	age	Gender	ASA	smoker	Admission reason	Timing of implant displacement	Immediate implant	Sinus elevation	OAF	Method of OAF closure	ostium obstruction	sinusitis	method of implant removal	Time to removal	Year of treatment	Sinus membrane thickening	Pus discharge	pain	complications	Implant length (mm)	sinus floor thickness (mm)
1	65	M	2	0	Pre prosthetic	NA	NA	0	0	0	1	0	FESS	1 year >	2023	1	0	0	0	NA	2.4
2	70	F	2	0	Dentist referral after displacement	Implantation	NA	0	0	0	0	1	FESS	1 year <	2022	1	1	1	0	11.5	1
3	57	M	3	1	Coincidental finding	NA	NA	0	0	0	1	1	FESS	1 year <	2023	1	1	0	0	10	1.8
4	33	F	1	0	Dentist referral after displacement	Implantation	1	0	0	0	1	0	FESS	1 year >	2022	1	0	0	0	6	2.5
5	67	F	1	0	Dental symptoms	Exposure	NA	0	0	0	1	1	FESS	1 year <	2020	1	1	0	0	11.5	2.9
6	65	M	2	1	Pre prosthetic	NA	NA	0	0	0	1	1	FESS	1 year <	2020	1	0	0	0	16	2.2
7	59	M	1	0	Dental symptoms	NA	NA	0	1	BFP	1	1	CALDWELL-LUC	1 year <	2015	1	1	0	0	NA	NA
8	71	M	2	1	Pre prosthetic	NA	NA	0	0	0	1	1	FESS	1 year <	2019	1	1	0	0	8	2.9
9	82	M	2	0	Dental symptoms	NA	NA	0	1	BFP	0	0	FESS	1 year <	2021	1	1	1	0	11.5	1.5
10	54	F	2	0	Dentist referral after displacement	Implantation	NA	0	1	BAF	1	1	FESS	1 year >	2020	1	0	1	0	13.5	3.1
11	68	M	2	0	Rhinologic symptoms	Implantation	1	1	1	BFP	1	1	FESS	1 year >	2020	1	1	1	0	13	2.9
12	56	M	2	0	Rhinologic symptoms	NA	0	0	1	BFP	1	1	FESS	1 year <	2022	1	1	0	0	NA	3.3
13	74	M	3	0	Pre prosthetic	NA	0	0	1	BFP	1	1	FESS	1 year >	2023	1	0	0	0	8	3.5
14	56	F	2	1	Pre prosthetic	Implantation	0	0	1	BAF + PALATAL FLAP	0	0	FESS	1 year <	2018	1	0	0	OAF	8	2.4
15	54	F	1	1	Dentist referral after displacement	Exposure	0	0	1	BAF	0	0	TRANSCRESTAL	1 year >	2021	1	0	0	0	NA	NA
16	68	M	1	0	Pre prosthetic	NA	0	0	0	0	1	1	FESS	1 year >	2021	1	1	1	0	10	1.8
17	19	F	1	0	Rhinologic symptoms	Implantation	1	0	1	BAF	0	0	TRANS CRESTAL	1 year >	2019	1	0	0	0	NA	NA
18	57	M	1	0	Rhinologic symptoms	Exposure	NA	0	0	0	0	0	FESS	1 year >	2018	1	1	0	0	NA	2.5
19	86	M	3	0	Dental symptoms	NA	NA	1	1	BFP	0	0	FESS	1 year >	2018	0	0	0	0	10	1.2
20	85	M	2	0	Pre prosthetic	NA	NA	1	0	BAF	0	0	LATERAL APPROACH	1 year >	2023	1	0	0	0	10	1
21	55	M	1	1	Dentist referral after displacement	NA	0	0	1	0	0	0	CALDWELL-LUC	1 year >	2011	1	1	0	0	NA	NA
22	68	M	3	0	Pre prosthetic	NA	0	1	1	BAF	1	0	CALDWELL-LUC	1 year >	2011	1	1	0	0	NA	NA
23	36	F	1	0	Rhinologic symptoms	Implantation	0	0	0	0	0	1	FESS	1 year >	2023	1	1	1	0	NA	1
24	85	M	2	0	Dentist referral after displacement	Osseointegration period	0	1	0	0	0	0	LATERAL APPROACH	1 year >	2010	1	0	0	0	10	1
25	56	M	2	1	Rhinologic symptoms	NA	NA	1	0	0	1	1	FESS	1 year <	2022	1	1	0	0	13	2.5
26	74	M	2	0	Rhinologic symptoms	NA	NA	1	0	0	1	1	FESS	1 year <	2023	1	1	1	0	8	1.5
27	61	F	1	0	Dentist referral after displacement	Exposure	NA	1	0	0	0	1	FESS	1 year >	2023	1	0	1	0	10	3.6
28	63	F	1	0	Coincidental finding	NA	NA	0	0	0	0	0	No treatment	No treatment needed	NA	0	0	0	0	10	3
29	44	F	3	1	Coincidental finding	Osseointegration period	0	0	0	0	0	0	CALDWELL-LUC	1 year >	2015	1	0	0	0	13	2
30	61	F	3	1	Dentist referral after displacement	Osseointegration period	0	1	0	0	0	0	No treatment	No treatment needed	NA	0	0	0	0	11.5	1
31	79	F	1	0	Rhinologic symptoms	NA	NA	0	0	0	1	1	SPONTANEOUS - SNEEZE	No treatment needed	NA	1	1	1	0	11.5	2
32	65	F	2	1	Pre prosthetic	NA	0	0	0	0	0	0	LATERAL APPROACH	1 year <	2023	0	0	0	0	13	3

*OAF– Ora-antral fistula; **BFP– Buccal fat pad; ***BAF– Buccal advancement flap

**Table 2:** Implant removal method of total cohort

Modality	*N*	%
Fess	20	63
Caldwell luc	4	13
Lateral approach	3	9
Trans crestal	2	6
No treatment	2	6
Spontaneous - Sneezing	1	3
**TOTAL**	**32**	**100**

## Data Availability

Data is provided within the manuscript or supplementary information files.

## Abbreviations

OAF: Oro-antral fistula
FESS: Functional endoscopic sinus surgery
OMC: Osteo-meatal complex
ODS: Odontogenic sinusitis

## References

[1] Galindo-Moreno P, Padial-Molina M, Avila G, Rios HF, Hernández-Cortés P, Wang HL. Complications associated with implant migration into the maxillary sinus cavity. Clin Oral Implants Res. 2012;23(10):1152–60. 22092923 10.1111/j.1600-0501.2011.02278.x

[2] Seigneur M, Hascoët E, Chaux AG, Lesclous P, Hoornaert A, Cloitre A. Characteristics and management of dental implants displaced into the maxillary sinus: a systematic review. Int J Oral Maxillofac Surg. 2023;52(2):245–54. 35778233 10.1016/j.ijom.2022.06.009

[3] Damlar İ. Disappearance of a dental implant after migration into the maxillary sinus: an unusual case. J Korean Assoc Oral Maxillofac Surg. 2015;41(5):278. 26568932 10.5125/jkaoms.2015.41.5.278 PMC4641221

[4] Sgaramella N, Tartaro G, D’Amato S, Santagata M, Colella G. Displacement of dental implants into the maxillary sinus: A retrospective study of Twenty-One patients. Clin Implant Dent Relat Res. 2016;18(1):62–72. 24889650 10.1111/cid.12244

[5] González-García A, González-García J, Diniz-Freitas M, García-García A, Bullón P. Accidental displacement and migration of endosseous implants into adjacent craniofacial structures: A review and update. Med Oral Patol Oral Cir Bucal. 2012;17(5). 10.4317/medoral.18032 PMC3482520 22549685

[6] Brescia G, Fusetti S, Apolloni F, Marioni G, Saia G. Displaced Dental Materials in the Maxillary Sinus: An Original Series. Analysis and Definition of a Surgical Decision-Making Process. Annals of Otology, Rhinology and Laryngology. 2019;128(3):177–83. 10.1177/0003489418812898 30461291

[7] Quiney RE, Brimble E, Hodge M. Maxillary sinusitis from dental osseointegrated implants. J Laryngol Otol [Internet]. 1990 [cited 2025 Mar 24];104(4):333–4. Available from: https://pubmed.ncbi.nlm.nih.gov/2370457/10.1017/s0022215100112630 2370457

[8] Felisati G, Lozza P, Chiapasco M, Borloni R. Endoscopic removal of an unusual foreign body in the sphenoid sinus: an oral implant. Clin Oral Implants Res [Internet]. 2007 Dec [cited 2025 Mar 24];18(6):776–80. Available from: https://pubmed.ncbi.nlm.nih.gov/17868385/10.1111/j.1600-0501.2007.01409.x 17868385

[9] Kim SM. The removal of an implant beneath the optic Canal by modified endoscopic-assisted sinus surgery. Eur Arch Otorhinolaryngol. 2017;274(2):1167–71. 27942890 10.1007/s00405-016-4416-4

[10] Jeong KI, Kim SG, Oh JS, You JS. Implants displaced into the maxillary sinus: A systematic review. Implant Dent. 2016;25(4):547–51. 26974033 10.1097/ID.0000000000000408

[11] Chiapasco M, Felisati G, Maccari A, Borloni R, Gatti F, Di Leo F. The management of complications following displacement of oral implants in the paranasal sinuses: a multicenter clinical report and proposed treatment protocols. Int J Oral Maxillofac Surg. 2009;38(12):1273–8. 19781911 10.1016/j.ijom.2009.09.001

[12] Laureti M, Ferrigno N, Rosella D, Papi P, Mencio F, De Angelis F et al. Unusual case of osseointegrated dental implant migration into maxillary sinus removed 12 years after insertion. Case Rep Dent. 2017;2017. 10.1155/2017/9634672 PMC5368374 28392949

[13] Hamdoon Z, Mahmood N, Talaat W, Sattar AA, Naeim K, Qais A et al. Evaluation of different surgical approaches to remove dental implants from the maxillary sinus. Sci Rep. 2021;11(1). 10.1038/s41598-021-83721-z PMC7904809 33627752

[14] Matti E, Emanuelli E, Pusateri A, Muniz CCS, Pagella F. Transnasal endoscopic removal of dental implants from the maxillary sinus. Int J Oral Maxillofac Implants. 2013;28(3):905–10. 23748326 10.11607/jomi.2894

[15] Varol A, Türker N, Göker K, Basa S. Endoscopic retrieval of dental implants from the maxillary sinus. Int J Oral Maxillofac Implants [Internet]. 2006 [cited 2025 Mar 24];21(5):801–4. Available from: https://pubmed.ncbi.nlm.nih.gov/17066644/17066644

[16] Craig JR, Poetker DM, Aksoy U, Allevi F, Biglioli F, Cha BY, et al. Diagnosing odontogenic sinusitis: an international multidisciplinary consensus statement. Int Forum Allergy Rhinol. 2021;11(8):1235–48. 33583151 10.1002/alr.22777

[17] Ridaura-Ruiz L, Figueiredo R, Guinot-Moya R, Piñera-Penalva M, Sanchez-Garcés MA, Valmaseda-Castellón E et al. Accidental displacement of dental implants into the maxillary sinus: A report of nine cases. Clin Implant Dent Relat Res. 2009;11(SUPPL. 1). 10.1111/j.1708-8208.2009.00175.x 19438955

[18] Petruson B. Sinuscopy in patients with titanium implants in the nose and sinuses. Scand J Plast Reconstr Surg Hand Surg [Internet]. 2004 [cited 2025 Mar 24];38(2):86–93. Available from: https://pubmed.ncbi.nlm.nih.gov/15202665/10.1080/02844310310023909 15202665

[19] De Groote MA, Fang FC. NO inhibitions: antimicrobial properties of nitric oxide. Clin Infect Dis [Internet]. 1995 [cited 2025 Mar 25];21 Suppl 2. Available from: https://pubmed.ncbi.nlm.nih.gov/8845445/10.1093/clinids/21.supplement_2.s162 8845445

[20] Manor Y, Anavi Y, Gershonovitch R, Lorean A, Mijiritsky E. Complications and management of implants migrated into the maxillary sinus. Int J Periodontics Restor Dent. 2018;38(6):e112–8. 10.11607/prd.3328 29897353

[21] Peleg M, Mazor Z, Chaushu G, Garg AK. Sinus floor augmentation with simultaneous implant placement in the severely atrophic maxilla. J Periodontol. 1998;69(12):1397–403. 9926770 10.1902/jop.1998.69.12.1397

[22] Lundgren S, Cricchio G, Hallman M, Jungner M, Rasmusson L, Sennerby L. Sinus floor elevation procedures to enable implant placement and integration: techniques, biological aspects and clinical outcomes. Periodontol 2000. 2017;73(1):103–20. 28000271 10.1111/prd.12165

[23] Lie SAN, Claessen RMMA, Leung CAW, Merten HA, Kessler PAWH. Non-grafted versus grafted sinus lift procedures for implantation in the atrophic maxilla: a systematic review and meta-analysis of randomized controlled trials. Int J Oral Maxillofac Surg. 2022;51(1):122–32. Available from: https://www.ijoms.com/action/showFullText?pii=S0901502721001260 10.1016/j.ijom.2021.03.016 33849784

[24] Kluppel LE, Santos SE, Olate S, Filho FWVF, Moreira RWF, de Moraes M. Implant migration into maxillary sinus: description of two asymptomatic cases. Oral Maxillofac Surg. 2010;14(1):63–6. 19865837 10.1007/s10006-009-0184-2

[25] Kim YK, Hwang JY, Yun PY. Relationship between prognosis of dental implants and maxillary sinusitis associated with the sinus elevation procedure. Int J Oral Maxillofac Implants. 2013;28(1):178–83. 23377064 10.11607/jomi.2739

[26] Fugazzotto P, Melnick PR, Al-Sabbagh M. Complications when augmenting the posterior maxilla. Dent Clin North Am. 2015;59(1):97–130. 25434561 10.1016/j.cden.2014.09.005

[27] Raghoebar GM, Vissink A. Treatment for an endosseous implant migrated into the maxillary sinus not causing maxillary sinusitis. Case Report; 2003. 14579964

[28] Regev E, Smith RA, Perrott D, Ma P. Maxillary sinus complications related to endosseous implants. Int J Oral Maxillofac Implants. 1995. 7672848

[29] Cummings G, Flint PW. Cummings otolaryngology: head and neck surgery. 2015.

[30] Scarano A, Perrotti V, Carinci F, Shibli JA. Removal of a migrated dental implant from the maxillary sinus after 7 years: a case report. Oral Maxillofac Surg [Internet]. 2011 Dec [cited 2025 Mar 24];15(4):239–43. Available from: https://pubmed.ncbi.nlm.nih.gov/20676909/10.1007/s10006-010-0243-8 20676909

[31] Borgonovo A, Fabbri A, Boninsegna R, Dolci M, Censi R. Displacement of a dental implant into the maxillary sinus: case series. Minerva Stomatol. 2010;59(1–2):45–54. 20212409

[32] Tatum H. Maxillary and sinus implant reconstructions. Dent Clin North Am. 1986;30(2):207–29. 3516738

[33] Stammberger H, Posawetz W. Functional endoscopic sinus surgery - Concept, indications and results of the Messerklinger technique. Eur Arch Otorhinolaryngol. 1990;247(2):63–76. 2180446 10.1007/BF00183169

[34] Kitamura A. Removal of a migrated dental implant from a maxillary sinus by transnasal endoscopy. Br J Oral Maxillofac Surg. 2007;45(5):410–1. Available from: https://pubmed.ncbi.nlm.nih.gov/16457911/10.1016/j.bjoms.2005.12.007 16457911

[35] Kraut RA, Smith RV. Team approach for closure of Oroantral and oronasal fistulae. Atlas Oral Maxillofacial Surg Clin. 2000;8(1):55–75. 11212387

[36] Parvini P, Obreja K, Begic A, Schwarz F, Becker J, Sader R et al. Decision-making in closure of Oroantral communication and fistula. Int J Implant Dent. 2019;5(1). 10.1186/s40729-019-0165-7 PMC6441669 30931487

